# Otoacoustic emissions in neonates exposed to smoke during pregnancy

**DOI:** 10.1016/j.bjorl.2019.08.001

**Published:** 2019-09-17

**Authors:** Alessandra Spada Durante, Cristina Moraes do Nascimento, Cristiane Lopes

**Affiliations:** Santa Casa de São Paulo, Escola de Ciências Médicas, São Paulo, SP, Brazil

**Keywords:** Otoacoustic emissions, spontaneous, Infant, newborn, Cochlea, Tobacco, Hair cells, auditory

## Abstract

**Introduction:**

The toxic substances present in cigarette smoke can damage cochlea hair cells. This effect has been investigated by measuring otoacoustic emissions.

**Objective:**

To investigate the impact of stimuli on otoacoustic emissions, comparing neonates with and without exposure to cigarette smoke during pregnancy.

**Methods:**

Transient-evoked otoacoustic emissions, evoked by a click stimulus, and distortion product otoacoustic emissions, evoked by two tones tests were conducted in both ears, using an Interacoustic TITAN device. The study included 105 neonates divided into two groups: a study group, comprising 47 neonates exposed to smoke during pregnancy; and a control group comprized of 58 neonates who were not exposed. All participants had normal neonatal hearing screening.

**Results:**

No statistical differences in distortion product otoacoustic emissions response levels were found between the groups. In the transient-evoked otoacoustic emissions tests lower response levels were observed in the study group than the control group in frequency band analysis of the right ear, with statistically significant differences in signals and signal-noise ratio (except at 1 kHz).

**Conclusion:**

The impact of smoking exposure could be analyzed through transient-evoked otoacoustic emissions in neonates. The group effect of smoke exposure during pregnancy was evidenced by a reduction in transient-evoked otoacoustic emissions levels. This same effect was not observed for the analyses performed on distortion product otoacoustic emissions levels.

## Introduction

Passive smoking is defined as inhalation of cigarette smoke present in the air. In most countries, an estimated 4.4-%‒8.4% of the population, including children, is exposed to passive smoking in the domestic environment. An estimated 600,000 people die every year from diseases secondary to passive exposure to tobacco smoke.^1^

Passive smoking is especially harmful during pregnancy, negatively impacting fetal growth. Toxic substances present in the composition of cigarettes, such as arsenic, lead and mercury, readily pass through the placenta and affect the fetus. These toxic elements cause changes in metabolism, reducing the supply of nutrients and oxygen to the fetus and, in the auditory system, can damage cochlea hair cells.^1^, ^2^, ^3^, ^4^

The impact of toxic substances on outer hair cells of the cochlear can be measured using Otoacoustic Emissions (OAE). These emissions are low intensity sounds produced spontaneously from vibrations generated by the cochlea, but can also be evoked by acoustic stimuli, which reflect inner ear health and can be measured in an objective, rapid and non-invasive manner. Smokers and individuals exposed passively to cigarette smoke exhibit lower otoacoustic emission responses.^5^, ^6^, ^7^, ^8^, ^9^ Simultaneous exposure to noise and carbon monoxide studied in Long-Evans rats induced large shifts in pure tone thresholds at all frequencies.^10^ Widespread hair cell loss was also seen, with outer hair cells appearing to be particularly vulnerable. These data add to a growing body of evidence showing that hearing loss after exposure to tobacco smoke may be correlated with metabolic insufficiency.^11^

Besides these traditional methods of classifying OAE based on the stimulus used,^12^ another approach classifies the OAE based on the generation mechanisms: distortion and/or reflection.^13^ In TEOAE the main mechanism appears to be reflection, while DPOAE is recognized to be a vector sum of the two major components, distortion and the reflection.^14^, ^15^, ^16^, ^17^

Several studies have investigated the influence of smoke exposure on OAE. However, the impact of smoking on Otoacoustic Emission (OAE) levels, according to the type of stimulus, is not yet known. The purpose of the present study is to measure the impact of the stimuli on Otoacoustic Emission (OAE), comparing neonates with and without exposure to cigarette smoke during pregnancy.

## Methods

A cross-sectional study was carried out at the rooming-in ward of the Santa Casa de Misericordia de São Paulo from June 2015 to August 2016, after approval by the Research Ethics Committee (permit 771.404).

### Sample

The sample was comprised of 105 neonates at 24–72 hours after birth, selected randomly. Subjects were divided into two groups. The Study Group (SG) of 47 neonates exposed to smoke during pregnancy comprised 21 boys and 26 girls. The Control Group (CG) of 58 neonates with no exposure to smoking during pregnancy comprised 35 boys and 23 girls. The division of the groups was based on information reported by the mothers regarding their smoking habits.

The inclusion and exclusion criteria were described in Table 1.

**Table 1:** Inclusion and exclusion criteria for the study.

Criteria		Control group	Study group
Inclusion	Mothers with no history of alcohol and/or drugs use during gestational period, and neonates with no risk factors for hearing loss and with normal results on neonatal auditory screening i.e. presence of evoked transient otoacoustic emissions measured on an automated response analysis.	+	+
	Neonates exposed to tobacco smoke because their mothers were active smokers throughout pregnancy.	–	+
	Neonates whose mothers were not exposed to passive smoking during pregnancy.	+	–
Exclusion	Not having ideal conditions for full application of the study protocol post-partum.	+	+

All procedures (select participants, perform the tests and refer participants for interventions) were performed by the same audiologist.

### Equipment

A Titan device (Interacoustics) with modules was used:

DPOAE: Two primary tone stimuli f_1_ and f_2_ were produced at f_2_ frequencies of 2, 3, 4, 5, 6 and 8 kHz, f_1_/f_2_ ratio = 1.22 at intensities of 65 and 55 dB SPL, for f_1_ and f_2_, respectively. The distortion product recorded and analyzed was 2f_1_–f_2_.

TEOAE: A wideband, non-linear, click stimulus (frequency range 500–5000 Hz) at an intensity of 80 dB peSPL was produced. Responses were analyzed for total response and also by frequency bands 1, 2, 3, 4 and 5 kHz (Fast Fourier Transform).

### Study protocol

Anamnesis was conducted with the mothers to investigate smoking habits: cigarette use, first use, number of cigarettes per day in the trimesters of pregnancy and on the day of delivery, and passive exposure.

DPOAE and TEOAE were recorded in a quiet, non-sound proofed room, during the post-partum stay (24–72 h). The tests were conducted only when the neonates were in natural sleep and the environment was quiet.

### Data analyses

Differences in DPOAE and TEOAE obtained in both ears were compared between the control and study groups at each frequency by Student´s *t*-test. The level of statistical significance adopted was *p* ≤ 0.05. Subsequently, the D’Agostino-Pearson normality test was conducted. All data passed the normality test (α > 0.05, data not shown).

All analysis was performed using GraphPad Prism 5.0 software.

## Results

**Figure 1:**
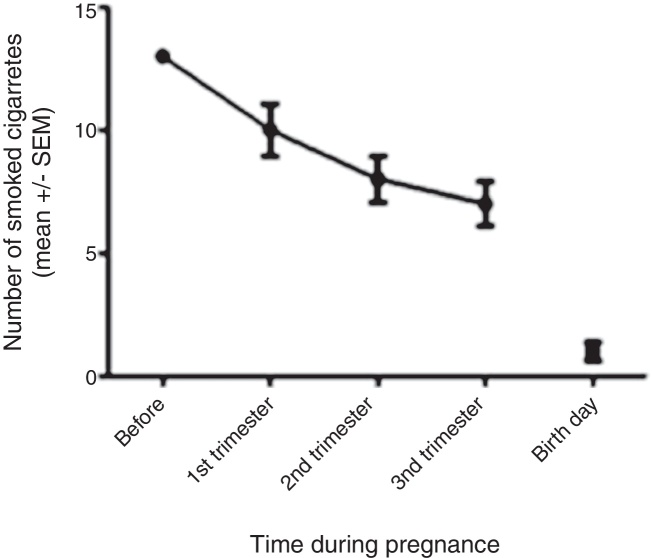
Cigarette consumption during pregnancy. Number of cigarettes smoked by the 47 maternal smokers before and during the different trimesters of pregnancy. Data expressed as mean ± SEM.

The number of cigarettes smoked by the mothers before and during pregnancy is given in Fig. 1. The maternal smokers in this study reduced the number of cigarettes smoked during the course of the pregnancy to almost zero on the day of delivery.

The perinatal characteristics of the neonates and mothers can be found in the descriptive data given in Table 2. All of the variables studied were similar in the two groups assessed, except for lower weight and height of the neonates with exposure to smoking during pregnancy.

**Table 2:** Perinatal characteristics of neonates and mothers in maternal smoker and non-smoker groups.

Variable		Group	Mean	Median	SD	Minimum	Maximum	*p*-value
Neonate	Gestational age (weeks)	Control	39	39	1.3	36	41	0.188
		Study	39	39	1.3	36	41	
	Weight (g)	Control	3.320	3.358	312.6	2.465	3.925	0.027*
		Study	3.152	3.045	425.2	2.26	4.215	
	Height (cm)	Control	48.6	49	2.3	3.7	53	0.039*
		Study	48.1	48	1.8	42	52.3	
	Apgar	Control	9	9	0.6	7.1	9.9	0.897
		Study	9	9	1.8	0	10.1	
	Head circumference (cm)	Control	34.23	34	1.522	31	37	0.696
		Study	34.12	34	1.514	30	37	
Mother	Age (years)	Control	27	28	6.9	15	42	0.503
		Study	28	28	7.7	16	44	
	Nº of consultations	Control	7	8	4.0	0	14	0.672
		Study	7	8	4.2	0	20	

* p < 0.05

The assessments of the cochlear physiology using Distortion Product Otoacoustic Emissions (DPOAE) and evoked Transient Otoacoustic Emissions (TEOAE) were carried out in exposed neonates (Study Group) and unexposed neonates (Control Group).

**Figure 2:**
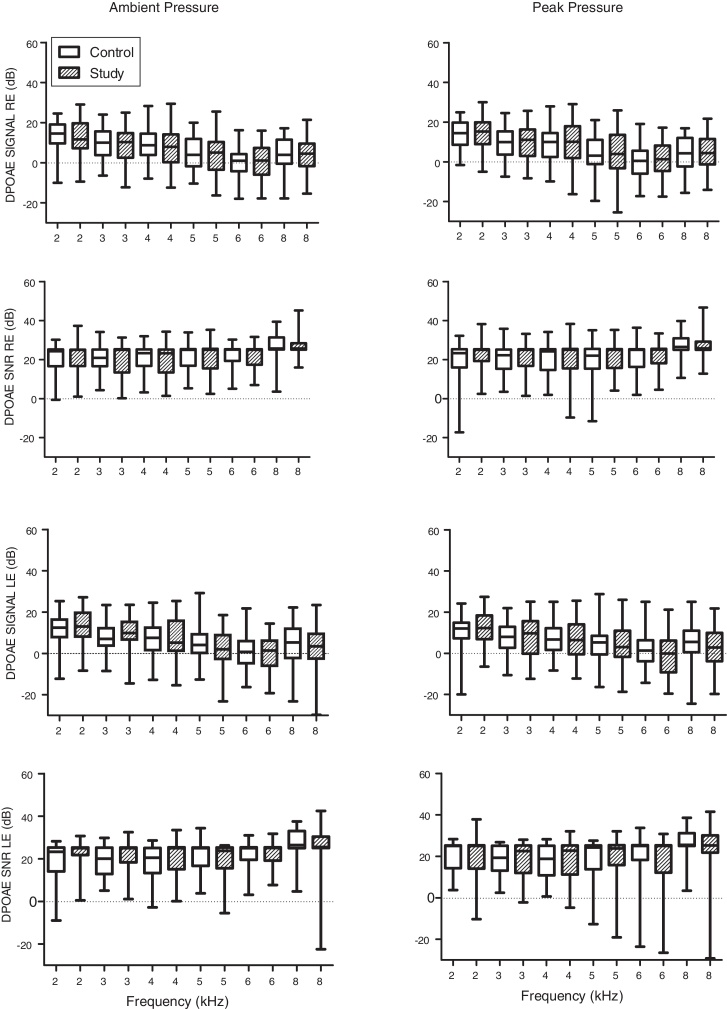
Box-plot of DPOAE signal and Signal/Noise Ratio (SNR) levels measured by group for Right Ear (RE) and Left Ear (LE). Control group represented by hollow rectangles and study group by hatched rectangles. No statistical differences were evident between the groups.

The mean response levels of the DPOAE signal and Signal/Noise Ratio (SNR) for the frequencies 2, 3, 4, 5, 6 and 8 kHz for both the right and left ears are depicted in Fig. 2. No statistical differences in DPOAE response levels were found between the groups. However, lower levels for the DPOAE signal measured in the right ear were evident in the study group at all frequencies tested.

**Figure 3:**
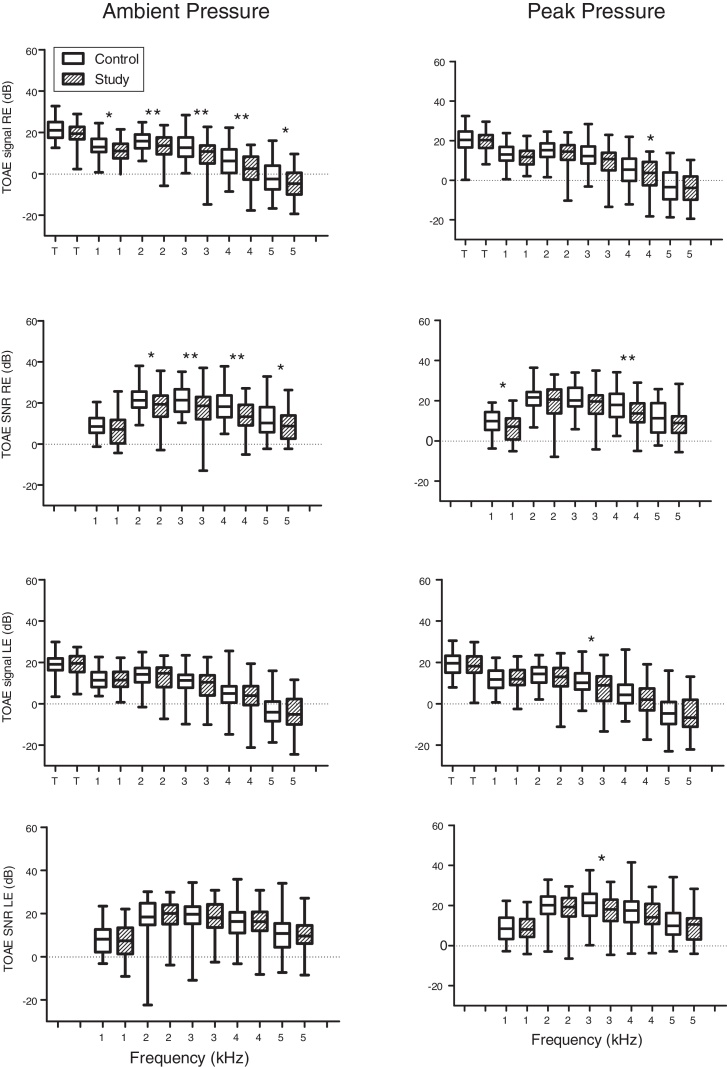
Box-plot of TOAE signal and Signal/Noise Ratio (SNR) levels measured by group for Right Ear (RE) and Left Ear (LE). Control group represented by hollow rectangles and study group by hatched rectangles. Asterisks indicate statistical comparison between control and study groups by frequency. One asterisk for *p* < 0.05 and two for *p* < 0.01.

The mean response levels of the TEOAE signal and signal/noise ratio for total response and the frequency bands 1, 2, 3, 4 and 5 kHz for the control and study groups in both the Right Ear (RE) and Left Ear (LE) are shown in Fig. 3.

Lower response levels were observed in the study group than the control group in the TEOAE frequency band analysis of the right ear, with statistically significant differences in the signals and signal-noise ratio (except 1 kHz).

## Discussion

Studies in newborns have demonstrated the harmful effect of exposure to smoke during pregnancy on the auditory system of neonates, showing that it leads to a reduction in TEOAE response levels in exposed compared to unexposed individuals, irrespective of the degree of exposure.^5^, ^7^

The importance of studying neonates exposed to smoking lies in the high rate of maternal smokers and the health impact of this exposure. It is estimated that, between 2005 and 2015, over half of the world population (2.8 billion people) was exposed to at least one antismoking protection measure.^18^ With the exception of African regions and the Eastern Mediterranean, the prevalence of smoking is declining globally. However, the number of smokers in the population remains high. Around 21% of the world´s population aged 15 or older (about 1.1 billion people) are smokers – approximately 35% men and 6% women. Tobacco is a highly addictive substance and the vast majority of users smoke daily.^1^

In the sample of maternal smokers assessed (study group), a reduction in smoking habit during the course of the pregnancy was observed (Fig. 1), a finding consistent with data in the literature.^1^ A marked decrease in cigarette use was evident on the day of delivery, close to zero, most likely due to the smoking restriction imposed by hospitalization. The blood collected from mothers´ umbilical cords showed similar concentrations of cotinine (a marker of nicotine use in the preceding 8–24 h) in both exposed and unexposed groups. Given that all the mothers had their babies by vaginal delivery, with labor lasting several hours, this result is in line with the reported number of cigarettes smoked on the day of delivery, but does not show chronic exposure of the fetus to nicotine throughout the gestational period. Therefore, the division of the groups was based only on the information reported by the mothers regarding their smoking habit, using the method validated by Caraballo et al.^19^

The association between smoking and harmful effects on the health of women, pregnant mothers and the fetus has been clearly established.^2^, ^3^, ^4^ The association between smoking during pregnancy and low birth weight and length has also been described.^3^ In our study, newborns of maternal smokers had body mass and height that were, on average, 168 g and 0.5 cm lower, respectively (*p* = 0.03 for both).

The impact of smoking on audiological responses has been explored in the literature in the population of adult smokers^20^, ^21^, ^22^, ^23^, ^24^ passively exposed adolescents,^25^ and passively exposed children,^5^, ^6^, ^8^, ^25^ revealing changes in responses in audiologic assessment batteries as a result of exposure to active and passive smoking.

The agents contained in tobacco can reduce and/or deplete oxygen levels to the cochlea, explaining the reduced amplitude of OAEs,^25^ measurable by assessment of the outer hair cells, an objective, non-invasive, sensitive and rapid method.

The main results of the present study revealed differences in TEOAE response levels that were found for several frequencies in both the left and right ears between the control and study groups, with statistically significant differences in signals and signal to noise ratio (except at 1 kHz). These findings are consistent with that of the Durante et al.^5^ study and are different from that of Korres et al.^7^ who found significant differences in TEOAEs at 4 kHz only.

The large intersubjective variations in the TOAE levels of the present study do not allow the identification of the effects of smoking in an individual neonate, but emphasize the value in TOAE measures in the research about the effect of secondhand smoke.

In contrast, the results obtained with DPOAE revealed no effect of exposure to smoke during pregnancy (Fig. 2). However, the first graph in Fig. 2 shows consistently lower DPOAE signal response levels in the right ear of the study group compared with the control group.

Only one intensity level was used with each method, TEOAE and DPOAE. Applying different intensities and configurations of stimulation affect the sensitivity of the test to cochlear pathology and probably smoking. In addition, the mechanism of OAE generation by distortion and/or reflection may be a relevant factor.^13^ Clinically obtained emissions are possibly a mix of two types of emissions, where the distortion mechanism is greater in DPOAE and reflection is greater in TEOAE.^14^, ^15^, ^16^, ^17^ A recent study suggests that the reflection component is more vulnerable to ototoxic insults,^26^ which might explain results in the literature showing that the effect of passive smoking on otoacoustic emissions of neonates is more evident in TEOAE, irrespective of degree of exposure.^6^, ^7^ The studies that found reduced DPOAE response levels were conducted in active smokers.^24^, ^25^, ^26^ Corroborating these findings, the results of the present study showed the effect of exposure to smoke during pregnancy on TEOAE measurements in neonates (Fig. 3).

The contribution of this study was to investigate the TEOAE and DPOAE in the same neonates, thus revealing the possibility of a different relationship between the generation mechanisms in the human cochlea and the impact of smoking exposure. Future studies of OAE should be conducted to investigate the relative magnitudes of the two DPOAE components in order to provide a better understanding of the underlying mechanisms of DPOAE production following smoking exposure.

## Conclusion

The impact of smoking exposure on the cochlea could be detected in neonates using TEOAE hearing screening data. In contrast DPOAE screening data from the same population did not demonstrate a significant reduction.

## Funding

This work was supported by the São Paulo Research Foundation - FAPESP [grant n^o^ 2014/15810-0].

## Conflicts of interest

The authors declare no conflicts of interest.
